# Candidate gene discovery for salt tolerance in rice (*Oryza sativa* L.) at the germination stage based on genome-wide association study

**DOI:** 10.3389/fpls.2022.1010654

**Published:** 2022-11-01

**Authors:** Chunyan Ju, Xiaoding Ma, Bing Han, Wei Zhang, Zhengwu Zhao, Leiyue Geng, Di Cui, Longzhi Han

**Affiliations:** ^1^ Chongqing Engineering Research Center of Specialty Crop Resources, College of Life Sciences, Chongqing Normal University, Chongqing, China; ^2^ Institute of Crop Sciences, Chinese Academy of Agricultural Sciences, Beijing, China; ^3^ Institute of Coastal Agriculture, Hebei Academy of Agriculture and Forestry Sciences, Tangshan, China; ^4^ Tangshan Key Laboratory of Rice Breeding, Tangshan, China

**Keywords:** rice, germination stage, salt stress, genetic structure, GWAS, candidate gene

## Abstract

Salt stress affects rice seed germination and seedling formation, seriously restricting rice production. Screening salt-tolerant rice varieties and analyzing the genetic mechanisms underlying salt tolerance are therefore very important to ensure rice production. In this study, 313 *Oryza sativa* ssp. *japonica* germplasm were used to conduct a genome-wide association study (GWAS) using 1% NaCl as a salt stress treatment during germination stage. The germination potential (GP) on different days and the germination index (GI) under salt stress were used as salt tolerance indicators. The results of population structure analysis showed that the 313 germplasm studied could be divided into two subpopulations, consistent with the geographical origins of the materials. There were 52 loci significantly related to salt tolerance during germination, and the phenotypic contribution rate of 29 loci was > 10%. A region on chromosome 11 (17049672–17249672 bp) was repeatedly located, and the candidate gene *LOC_Os11g29490*, which encodes a plasma membrane ATPase, was identified in this locus. Further haplotype analysis showed the GP of germplasm with different haplotypes at that locus significantly differed under salt stress (*p* < 0.05), and germplasm carrying Hap2 displayed strong salt tolerance during the germination stage. Two other promising candidate genes for salt tolerance were identified: *LOC_Os01g27170* (*OsHAK3*), which encodes a potassium transporter, and *LOC_Os10g42550* (*OsITPK5*), which encodes an inositol 1, 3, 4-trisphosphate 5/6-kinase. The results of this study provide a theoretical basis for salt-tolerant gene cloning and molecular design breeding in rice.

## Introduction

Approximately 30% of the rice-growing area in the world is affected by salt damage (Prasad et al., 2000; Takehisa et al., 2004). Data from the United Nations Science, Education and Food and Agriculture Organization show that China has 9,913 hectares of salinized land (He and Luo, 2021). Salt stress is one of the main abiotic stress factors that restricts stable rice production (Hu et al., 2012). Salt stress mainly causes osmotic stress, ion toxicity, and nutrient deficiency, which ultimately lead to yield reduction (Yang and Guo, 2018a; Yang and Guo, 2018b). Grattan et al. (2002) showed that the presence of 0.37% salt content caused a 50% decrease in rice yield. Therefore, screening salt-tolerant rice varieties and analyzing the genetic mechanisms of salt tolerance in rice are very important to ensure adequate rice production.

Salt tolerance in rice is a quantitative trait controlled by multiple genes and is susceptible to environmental factors (Roy et al., 2014). In recent years, quantitative trait locus (QTL) and association analyses have identified many QTLs for salt tolerance (Liu et al., 2019; Yuan et al., 2020; Kong et al., 2021; Nakhla et al., 2021; Nayyeripasand et al., 2021). Bonilla et al. (2002) used a recombinant inbred line population (F_8_) constructed from ‘IR29’ and ‘Pokkali’. They located *Saltol* between chromosome 1 RM23 and RM140 that accounted for 39.2%, 43.9%, and 43.2% of the total phenotypic variation in Na^+^ content, K^+^ content, and the Na^+^/K^+^ ratio, respectively. Wang et al. (2011) studied 150 recombinant inbred lines (RILs) (F_2:9_) generated by crossing the salt-tolerant *japonica* landrace ‘Jiucaiqing’ and the salt-sensitive *indica* variety ‘IR26’. They detected 16 QTLs related to imbibition rate and germination potential under 100 mM NaCl stress at the germination stage. Zeng et al. (2021) used a BC_1_F_2_ population constructed from ‘Wujiaozhan’ (‘WJZ’) and ‘Nipponbare’ (‘Nip’). They located nine QTLs related to germination rate and germination index under 300 mM NaCl salt stress at the germination stage; the main QTL *qGR6.2* was finely mapped to a 65.9-kb region. Shi et al. (2017) used 478 rice materials as associated groups. From a total of 6,361,920 single nucleotide polymorphisms (SNPs), they identified 22 that were significantly related to salt tolerance using the stress susceptibility index (SSIS) of vigor index (VI) and mean germination time (MGT) as salt tolerance evaluation indexes. Yu et al. (2018) used 1.65 million SNPs or insertion/deletion mutations (indels) to conduct a GWAS with 295 rice varieties at the germination stage, and identified 17 genes that may be related to salt tolerance. Naveed et al. (2018) used 395,553 SNP markers to carry out GWAS on 208 rice varieties, identifying six quantitative trait nucleotides (QTNs) that affected salt tolerance at the germination stage and 14 at the seedling stage. Islam et al. (2022) used the 2.8 M high-density SNP genotype map generated by the 3000 Rice Genomes Project (3KRGP) to identify 21 sites associated with salinity stress during the seed germination stage.

Some major QTLs identified from QTL and association analyses have been cloned. Lin et al. (2004) used the F_2_ population derived from the salt-tolerant variety ‘Nona bokra’ and the salt-sensitive variety ‘Koshihikari’ to locate the main QTL *qSKC-1*, which controls K^+^ content in stems and leaves; this locus accounts for 40.1% of the total phenotypic variation. Ren et al. (2005) isolated a gene encoding a high-affinity potassium transporter (HKT), *SKC1* (*OsHKT1;5*), through map-based cloning. *SKC1* regulates the dynamic balance of K^+^/Na^+^ under salt stress. He et al. (2019) used chromosome segment replacement lines (CSSLs) derived from the *japonica* line ‘Jiucaiqing’ and the *indica* line ‘IR26’ to locate the main QTL *qSE3*, which promotes seed germination and seedling formation under salt stress. Through map-based cloning, they also isolated *OsHAK21*, which encodes a potassium transporter. In a GWAS, Campbell et al. (2017) used 390 rice germplasm to locate a QTL related to Na^+^ content in roots, *RNC4*, on chromosome 4. They identified a candidate gene, *OsHKT1;1*, which plays an important role in regulating Na^+^ content in rice roots. In addition, (Fukuda et al., 1999; Fukuda et al., 2004) cloned the Na^+^/H^+^ antiporter OsNHX1 in the vacuolar membrane of rice and found that *OsNHX1* overexpression improves salt tolerance in transgenic rice. Horie et al. (2001) isolated *OsHKT2;1*, which encodes a high-affinity sodium transporter. Wei et al. (2021) found that *OsHKT2;1* is located downstream of *OsPRR73* and controls salt tolerance in rice by regulating sodium ion homeostasis and active oxygen levels. Tian et al. (2021) found that *OsAKT2*/*OsK3.1* encodes a Shaker family potassium channel protein, which contributes to maintenance of the overall Na^+^/K^+^ homeostasis in plants under salt stress; it enhances salt tolerance in rice by regulating K^+^ redistribution. Some salt tolerance genes have also been obtained through reverse genetic studies, including *SNAC1*, *SNAC2*, *ZFP252*, *ZFP182*, and *OsNAP* (Hu et al., 2006, Hu et al., 2008; Xu et al., 2008; Huang et al., 2012b; Chen et al., 2014). So far, most of studies have focused on salt tolerance QTLs at the germination stage (http://gramene.org/). However, little is known about genetic mechanisms of salt tolerance at the germination stage, and there is a lack of salt-tolerant candidate genes available for molecular design breeding. Therefore, it is crucial to elucidate the genetic mechanisms of salt tolerance and identify salt-tolerant candidate genes at the germination stage.

In the present study, we used a GWAS approach with 313 temperate *japonica* germplasm and two traits: germination potential on multiple days and germination index under 1% NaCl stress at the germination stage. This allowed us to explore candidate salt tolerance genes and provide a theoretical basis for salt-tolerant gene cloning and molecular design breeding in rice.

## Materials and methods

### Materials

We selected 313 temperate *japonica* germplasm as the study population for GWAS. These comprised 27 germplasm from Heilongjiang, China; 82 from Jilin, China; 120 from Liaoning, China; and 80 from Japan (**Table S1**). The raw sequence data reported in this paper have been deposited in the National Genomics Data Center (NGDC), part of the China National Center for Bioinformation (CNCB), under accession codes CRA004238 (https://ngdc.cncb.ac.cn/). The average sequencing depth and average genome coverage of these data were 25.60× and 90.29%, respectively (Cui et al., 2022).

### Identification of salt tolerance at germination stage

For each rice line, 30 seeds were dried at 50°C for 2 days to break seed dormancy. The seeds were disinfected with 1.4% sodium hypochlorite solution for 15 minutes, then washed with distilled water three times. Seeds were placed in a petri dish with two layers of filter paper and 10 mL 1% NaCl solution was added. In the control, the filter paper was soaked with 10 mL distilled water. Seeds were incubated in a growth chamber at 30°C under dark and the solution was replaced every day to maintain the NaCl concentration and the distilled water volume. Seed germination was measured on days 3 through 7; germination was defined as occurring when the bud length reached half of the seed length. There were two independent replicates of this experiment. Germination potential (GP) and germination index (GI) were used as salt tolerance indexes. GI was calculated as follows:


GI= Σ (Gt/Tt)

(Wang et al., 2010)

where Gt is the number of germinated seeds on day t and Tt is the time corresponding to Gt in days. Gangyuan8 (a tolerant line) was used as a positive control in our study and Koshihikari (a sensitive line) was used as a negative control in our study (Lin et al., 2004).

### Statistical analysis of phenotypic data

Phenotypic variation analysis, correlation analysis, generalized heritability ( $h_{B}^{2}$ ), and Student’s *t*-tests were conducted in IBM SPSS v26. Among them, Student’s *t*-tests is mainly conducted on the GP between the treatment and the control, and the GP and GI among different subpopulations under salt stress. The diversity index (H’) was calculated as described by Xu et al. (2020) in Excel 2019. The GP on different days under salt stress was divided into 10 grades with a gradient of 10%, and the GI under salt stress was divided into six grades with a gradient of five. H’ was calculated for different characters using the improved Shannon-Wiener diversity index as follows:


H'=(−ΣPilnPi)/lnN

(Xu et al., 2020) 

where *P_i_* refers to the percentage of varieties in the i_th_ grade of a trait in all varieties and N refers to the total number of all varieties. A phenotypic frequency distribution map was drawn in R. Violin plots were generated with the ‘ggplot2’ (Wickham, 2016) package in R.

### Population structure and genetic diversity analysis

PLINK (v1.90) (Purcell et al., 2007) was used to obtain 65,025 SNPs with a sliding window using the command with “– indep-Pairwise 100 10 0.2”. ADMIXTURE (v1.3.0) (Alexander et al., 2009) was then used to calculate the population structure, and Evanno’s (Evanno et al., 2005) method was used to calculate ΔK. SNPs were selected using thresholds of minor allele frequency (MAF) > 0.05 and missing rate ≤ 0.2. PopLDdecay (Zhang et al., 2019) was used to calculate the genome-wide average linkage disequilibrium (LD). After genetic distance was calculated with PLINK (Purcell et al., 2007), Phylip (v3.697) (Retief, 2000) was used to construct a phylogenetic tree using the neighbor-joining method. GCTA (v1.26.0) (Yang et al., 2011) software was used to calculate kinship and perform principal component analysis (PCA). VCFtools (v0.1.13) (Danecek et al., 2011) was used to calculate genetic diversity (π) and genetic differentiation index (F_ST_). A partial Manhattan map and an LD heatmap were drawn with LDBlockShow (v1.40) (Dong et al., 2021).

### GWAS

The mixed linear model (MLM) method was used in tassel (v5.0) (Bradbury et al., 2007) to perform GWAS for GP on different days and GI under salt stress. There were 1,291,609 SNPs used for genotyping with MAF > 0.05 and missing rate ≤ 20%. Covariates were included based on the population structure at K = 2. The significance threshold (*p* = 1.12 × 10^-6^) of the GWAS was determined using Bonferroni correction based on the estimated effective number of independent SNPs (Li et al., 2012) and we have determined *p* < 1 × 10^-6^ as the GWAS threshold. A QTL interval was classified as the region 100 kb upstream and downstream of a significant SNP site. Candidate genes were then explored in that interval. The location results of GP on different days and GI under salt stress were completely overlapped/partially overlapped, which was co-location/overlap interval. Manhattan plots were generated using the ‘CMplot package’ (Yin, 2020) in R.

## Results

### Genetic population structure

**Figure 1:**
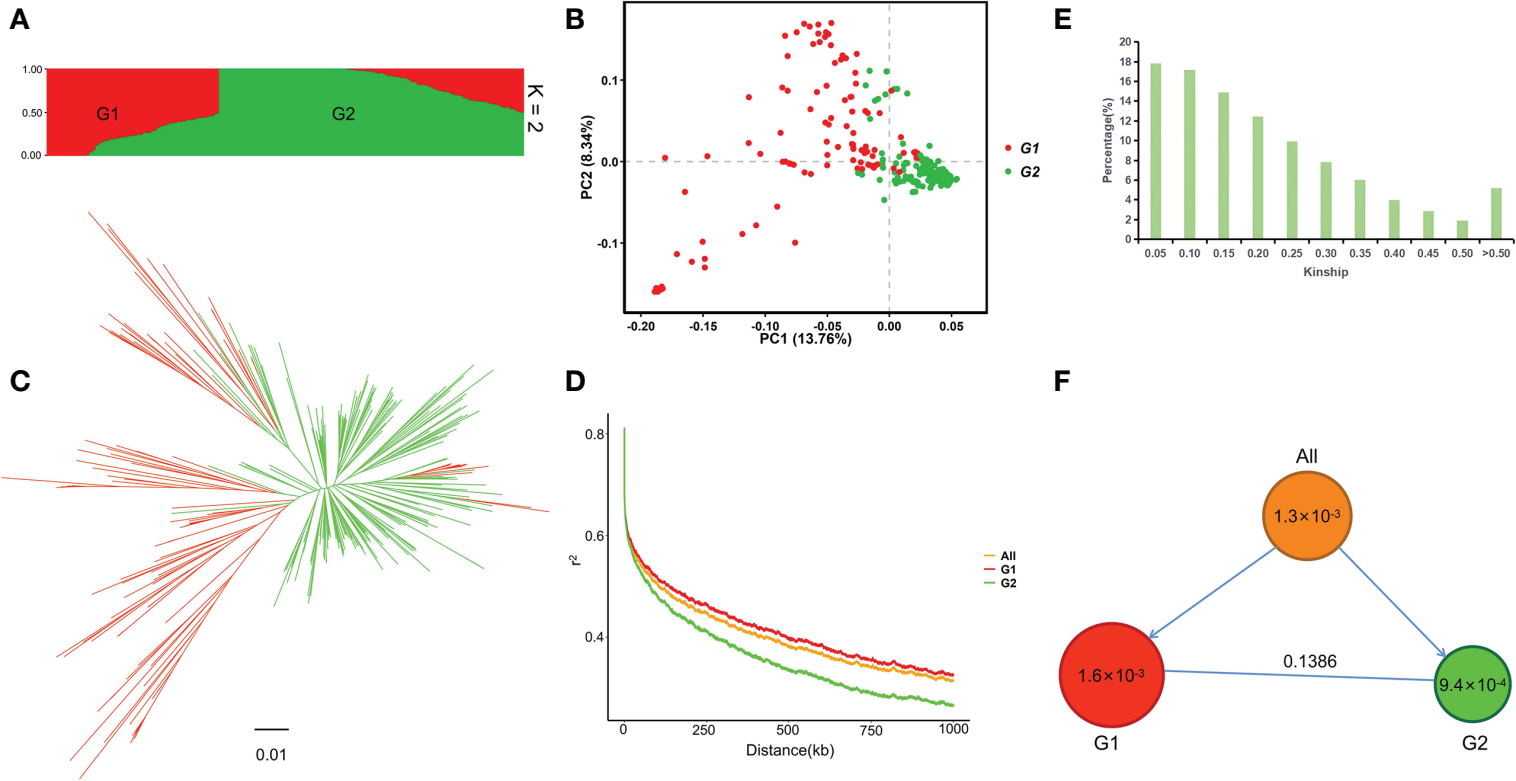
Population structure and genetic diversity in 313 *japonica* rice germplasm. **(A)** Population structure plot (K = 2). **(B)** PCA plot of the first two principal components. **(C)** Phylogenetic tree based on genetic distance. **(D)** Genome-wide average LD map of all germplasm (All) and the G1 and G2 subpopulations. **(E)** Kinship plot. **(F)** Genetic diversity and subpopulation differentiation of 313 germplasm. Circle size and the number within each circle represent the genetic diversity (π) of each group. The arrows indicate that all germplasm are divided into two subpopulations; the number on the straight horizontal line represents F_ST_ between the two subpopulations.

Population structure analysis showed that the maximum ΔK was at K = 2, so the 313 germplasm were divided into two subpopulations, named G1 (TeJ1) and G2 (TeJ2), respectively (**Figure S1**; **Figure 1A** and **Table S2**). G1 was composed of 113 materials, 103 of which were from Jilin and Liaoning, China. G2 was composed of 200 materials, most of which came from Japan and Heilongjiang, China. PCA showed that the first two principal components explained 22.10% of the genetic variation, and the germplasm in the G2 subpopulation were clustered significantly closer together than those in the G1 subpopulation (**Figure 1B**). The phylogenetic tree showed that the G1 and G2 subpopulations had significant genetic differentiation, consistent with the results of the population structure and PCA (**Figure 1C**). LD analysis showed that the LD attenuation distances of all germplasm (All), G1, and G2 were 399 kb, 489 kb, and 268 kb, respectively (**Figure 1D**). Kinship was > 0.3 for 19.95% of the materials, indicating that the genetic relationship between the studied materials was relatively close (**Figure 1E**). The genetic diversity of the G1 subpopulation (π = 1.6 × 10^-3^) was higher than that of the G2 subpopulation (π = 9.4 × 10^-4^). The F_ST_ between G1 and G2 was 0.1386 (**Figure 1F**).

### Phenotypic variation in the study population

**Figure 2:**
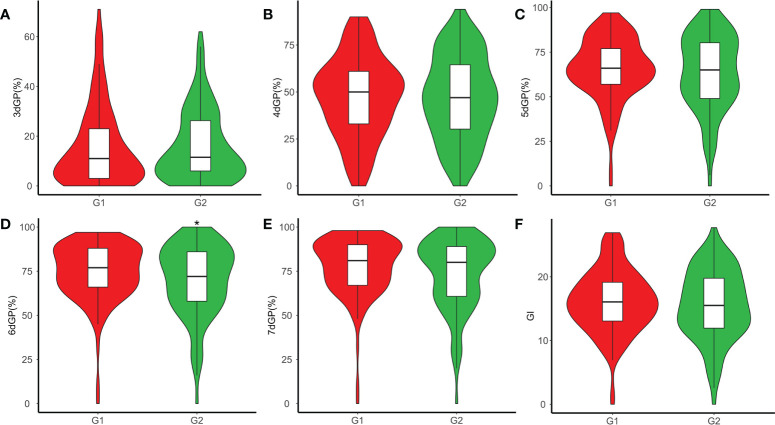
Phenotypic distribution of germination potential (GP) and germination index (GI) within subpopulations G1 and G2 under salt stress. **(A)** GP on day 3 (3dGP). **(B)** GP on day 4 (4dGP). **(C)** GP on day 5 (5dGP). **(D)** GP on day 6 (6dGP). **(E)** GP on day 7 (7dGP). **(F)** GI. *p < 0.05 (Student’s *t*-test).

In this study, we screened 313 accessions with germination rate of more than 90% for control condition and salt stress treatment, respectively. We found there was extremely significant difference of GP between control and days 3 through 7 of treatment (**Figure S2**). The GP across days and the GI under salt stress were used as evaluation indicators of salt tolerance for GWAS. The GP across days and the GI were continuously distributed under salt stress. (**Figures S3A-F**), demonstrating a typical quantitative character. The average GP values on days 3 through 7 under salt stress were 16.21%, 46.90%, 64.17%, 71.45%, and 74.82%, respectively (**Figure S4**). The mean GI value under salt stress was 15.77, with a range of 0–27.72. The coefficient of variation (CV) of GP on different days and GI under salt stress ranged from 25.39% to 92.54%. The CV of germination was highest on day 3 (92.54%) and lowest on day 7 (25.39%). Comparing the GP and GI under salt stress between G1 and G2 showed that the average GP value of G2 was significantly higher than that of G1 on day 6 (*p* < 0.05). There was no significant difference in GP on any other day or GI between the two subpopulations (**Figure 2**). The H’ of GP under salt stress was highest on day 4 (0.38), indicating that the phenotypic variation was the most abundant for that parameter. $h_{B}^{2}$ ranged from 78.74% to 98.09%. The $h_{B}^{2}$ of GP under salt stress was highest on day 3 (98.09%) and lowest on day 6 (78.74%) (**Table 1**). Correlation analysis showed that there was a very significant positive correlation between GP values on different days and between GP and GI under salt stress. The correlation coefficient was highest between GP on day 6 and GP on day 7 (r = 0.960**), followed by the correlation coefficient between GP on day 5 and GI (r = 0.947**). The lowest correlation coefficient was between GP on day 3 and GP on day 7 (r = 0.358**) (**Table 2**).

**Table 1:** Statistical analysis of germination potential (GP) on different days and germination index (GI) under salt stress.

Trait	Mean ± SD	Range	CV	H’	$h_{B}^{2}$
3dGP	16.21% ± 0.15	0%-71%	92.54%	0.25	98.09%
4dGP	46.90% ± 0.22	0%-94%	46.91%	0.38	84.73%
5dGP	64.17% ± 0.20	0%-99%	31.17%	0.35	79.83%
6dGP	71.45% ± 0.19	0%-100%	26.59%	0.34	78.74%
7dGP	74.82% ± 0.19	0%-100%	25.39%	0.32	79.65%
GI	15.77 ± 5.23	0-27.72	33.16%	0.26	82.06%

**Table 2:** Correlation analysis of germination potential (GP) on different days and germination index (GI) under salt stress.

	3dGP	4dGP	5dGP	6dGP	7dGP	GI
3dGP	1.000					
4dGP	0.776**	1.000				
5dGP	0.565**	0.856**	1.000			
6dGP	0.377**	0.649**	0.892**	1.000		
7dGP	0.358**	0.621**	0.872**	0.960**	1.000	
GI	0.774**	0.936**	0.947**	0.838**	0.818**	1.000

**p < 0.01.

### GWAS results

**Figure 3:**
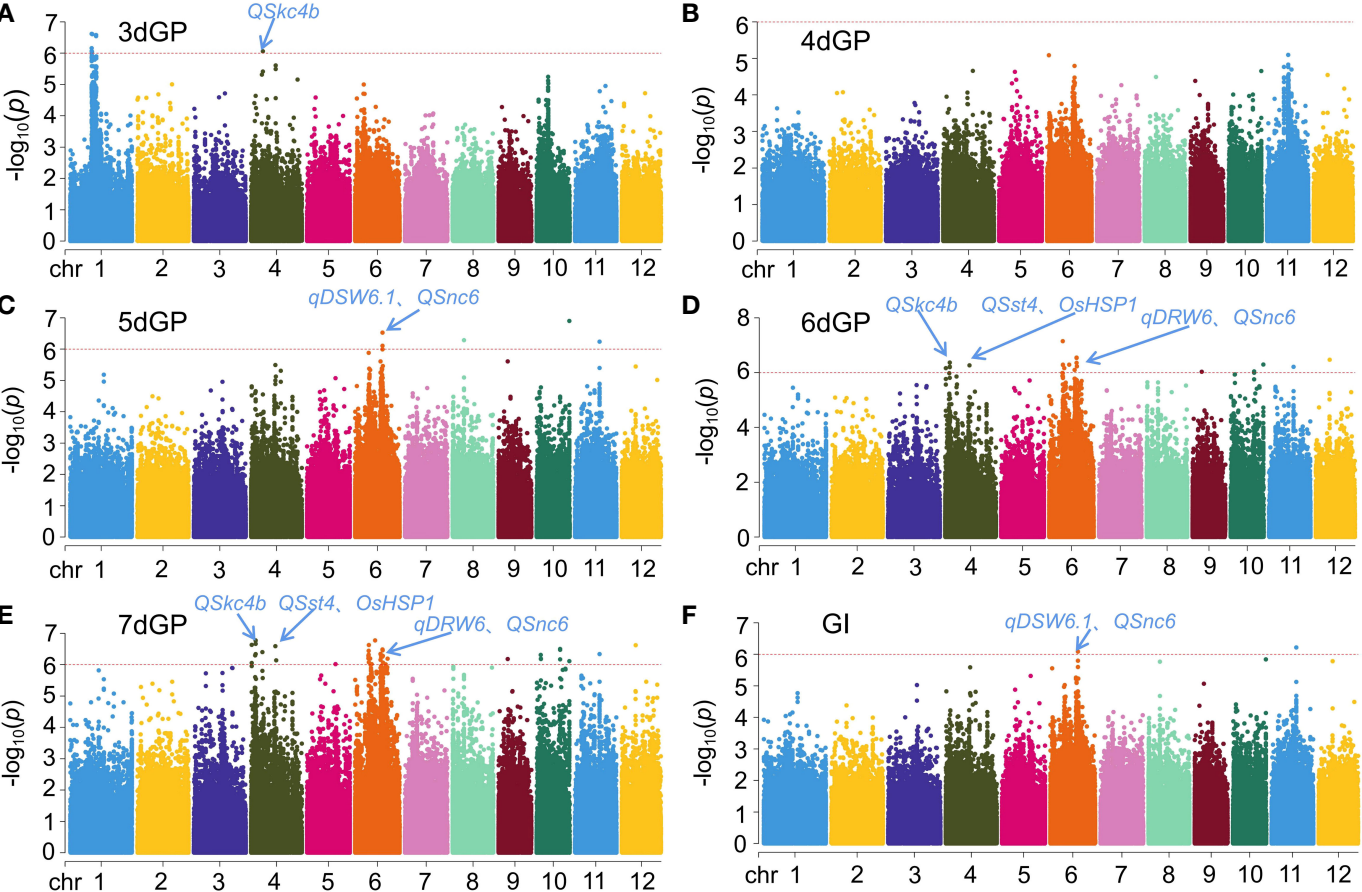
Results for GWAS of germination potential (GP) on different days and germination index **(GI)** under salt stress. **(A)** GP on day 3 (3dGP). **(B)** GP on day 4 (4dGP). **(C)** GP on day 5 (5dGP). **(D)** GP on day 6 (6dGP). **(E)** GP on day 7 (7dGP). **(F)** GI.

GWAS of GP on different days and GI under salt stress yielded 52 significant loci (*p* < 1 × 10^-6^) (**Figure 3** and **Table S3**). A total of five loci were located on chromosomes 1 and 4 that were significantly related to GP on day 3 (3dGP): *q3dGP1-1*, *q3dGP1-2*, *q3dGP1-3*, *q3dGP1-4*, and *q3dGP4*. The phenotypic contribution rates varied from 9.34% to 10.31%. There were four QTLs related to GP on day 5 (5dGP); the phenotypic contributions of *q5dGP6* and *q5dGP10* were largest, at 10.11% and 11.66%, respectively. There were 14 and 27 loci related to GP on day 6 (6dGP) and GP on day 7 (7dGP), respectively. Of those loci, there were six and 18, respectively, with phenotypic contribution rates > 10%. There were two loci discovered for GI, *qGI6* and *qGI11*, with phenotypic contributions of 9.37% and 10.00%, respectively. Co-location/overlap intervals were identified for GP on different days and for GI (**Table S3**). Specifically, chromosome 4 contained the co-loci *q6dGP4-1*/*q7dGP4-1* and *q6dGP4-3*/*q7dGP4-6*; chromosome 6 contained the co-loci *q6dGP6-2*/*q7dGP6-3*, *q6dGP6-3*/*q7dGP6-5*, and *q6dGP6-6*/*q7dGP6-9*; chromosome 9 contained the co-loci *q6dGP9*/*q7dGP9*; chromosome 10 contained the co-loci *q6dGP10-1*/*q7dGP10-4* and *q5dGP10*/*q6dGP10-2*/*q7dGP10-5*; chromosome 11 contained the co-loci *q5dGP11*/*q6dGP11*/*q7dGP11*/*qGI11*; chromosome 12 contained the co-loci *q6dGP12*/*q7dGP12*; chromosome 4 contained the overlapping loci *q6dGP4-2*/*q7dGP4-4*; and chromosome 6 contained the overlapping loci *q6dGP6-1*/*q7dGP6-2*, *q6dGP6-4*/*q7dGP6-6*, and *q5dGP6*/*q6dGP6-5*/*q7dGP6-8*/*qGI6* (**Table S3**).

### Discovery of candidate salt tolerance genes

**Figure 4:**
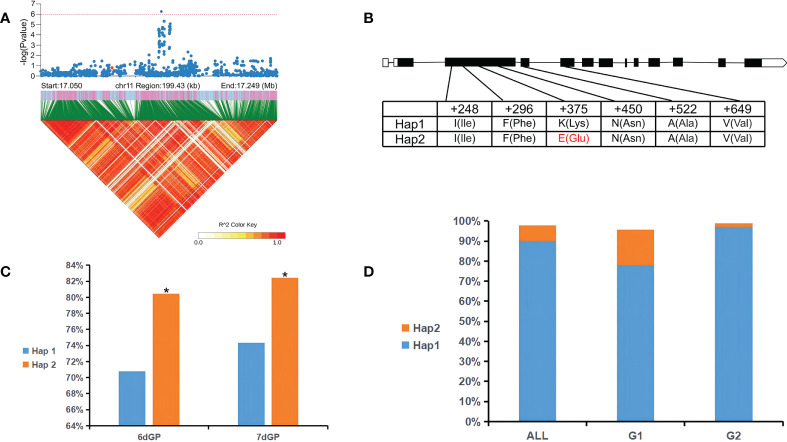
*LOC_Os11g29490* haplotype significance analysis. **(A)** Partial Manhattan map (top) and LD heatmap (bottom) around the peak of chromosome 11. The red dot indicates the position of a missense variation in *LOC_Os11g29490*. **(B)** Gene structure and polymorphism in *LOC_Os11g29490*. **(C)** Significance analysis of Hap1 and Hap2 among germination potential (GP) for all germplasm (All). **(D)** Distribution of Hap1 and Hap2 in All and in subpopulations G1 and G2. *p < 0.05 (Student’s *t*-test).

GWAS revealed a significant SNP (GP_Chr11_Pos17149672, *p* < 1 × 10^-6^) related to 5dGP, 6dGP, and 7dGP within a large LD region (> 300 kb) on chromosome 11 (17049672–17249672 bp) (**Figure 4A**). There were nine candidate genes with nonsynonymous mutations in this region. One was *LOC_Os11g29490*, which was 6,856 bp in length, containing 12 exons and encoding a plasma membrane ATPase. Previous studies have shown that the plasma membrane H^+^-ATPase is the key factor of SOS1 (an Na^+^/H^+^ antiporter) in response to salt stress (Yang et al., 2019). Further haplotype analysis showed that a nonsynonymous mutation (Lys→Glu) was due to an A→G substitution at site chr11:17109866, in an exon of *LOC_Os11g29490* (**Figure 4B**). This mutation could be used to divide the germplasm in this study into two haplotypes. There were significant (*p* < 0.05) differences in the GP between germplasm carrying the different haplotypes (**Figure 4C**). Hap2 was found to be the superior haplotype, and germplasm carrying Hap2 displayed strong salt tolerance during the germination stage. Hap2 accounted for 8% of all germplasm, but for 18% and 2% of G1 and G2, respectively (**Figure 4D**). This haplotype thus has great potential for breeding salt tolerance in rice.

The candidate gene *LOC_Os01g27170* (*OsHAK3*), which encodes a potassium transporter, was identified in a region on chromosome 10 containing loci for GP on days 5 through 7, and a homolog (*LOC_Os01g70490*/*OsHAK5*) has been shown to enhance salt stress tolerance in rice (Horie et al., 2011). *OsHAK3* is thus a promising candidate gene for salt tolerance. Similarly, a locus significantly related to 3dGP was identified on chromosome 1. The gene *LOC_Os10g42550* (*OsITPK5*) was present in this site and encodes inositol 1, 3, 4-trisphosphate 5/6-kinase; its homolog, *LOC_Os03g12840* (*DSM3*/*OsITPK2*), can enhance salt tolerance in rice (Du et al., 2011), making *OsTIPK5* a strong candidate for breeding salt tolerance (**Table 3**).

**Table 3:** Candidate genes related to salt stress at the germination stage.

Significant SNP locus	Physical Interval(bp)	MSU ID	Physical Interval(bp)	Function annotation
chr01_15145289	15045289-15245289	*LOC_Os01g27170*	15151960-15157245	Potassium transporter, putative, expressed
chr10_22899368	22799368-22999368	*LOC_Os10g42550*	22943495-22945212	Inositol 1, 3, 4-trisphosphate 5/6-kinase, putative, expressed
chr11_17149672	17049672-17249672	*LOC_Os11g29490*	17108744-17114115	Plasma membrane ATPase, putative, expressed

## Discussion

### Salt tolerance of rice during germination

Salt stress is one of the main abiotic stresses affecting rice yield (Wang et al., 2012; Liang et al., 2014; Shi et al., 2017; Ganie et al., 2019). In recent years, identification of salt tolerance genes and genetic analyses of salt tolerance traits in rice have attracted extensive attention from researchers (Mardani et al., 2014; Cui et al., 2015; Campbell et al., 2017; He et al., 2019). The effects of salt stress on rice seed germination mainly include osmotic stress and ion accumulation (Na^+^ and Cl^-^). In addition, the accumulation of Na^+^ leads to the imbalance of plant hormones, the generation of reactive oxygen species (ROS), and the change of cell membrane permeability (El Moukhtari et al., 2020). Because rice seed germination begins with water absorption, salinity prevents water absorption and ultimately inhibits seed germination (Othman et al., 2006). Wang et al. (2011) and Al-ansari and Ksiksi (2016) found that GP and GI are the best combinations for evaluation of salt tolerance phenotypes at the germination stage. In the present study, GP over several different days and GI in response to 1% NaCl stress were used as the evaluation indexes for salt tolerance. The genetic basis of salt tolerance was analyzed in rice at the germination stage using a population comprising 313 temperate *japonica* germplasm. In this study, we identified some salt-tolerant germplasm, such as Nonglin22, Liaogeng5, Shengnong265, Shangzhou, Churichu, and some salt-sensitive germplasm, such as Songgeng3, Fangzhu, Kendao10, Zhaori, Aoyu305.

### Population structure and genetic diversity

Population structure analysis in this study showed that the 313 rice germplasm could be divided into two subpopulations, G1 and G2. Members of the two groups clustered together in a manner consistent with the geographical origin of the materials and with the prior results of Cui et al. (2013). In this study, the LD decay distances we calculated in our study for all germplasm (All), G1, and G2 were 399 kb, 489 kb, and 268 kb, respectively, which were longer than that of a set of 809 *indica* rice accessions (Xie et al., 2015) and 3k rice population (Wang et al., 2018). The difference in LD decay distance between our investigation and the previous ones may be due to the possibly lower genetic diversity of rice accessions used in our study. Our findings indicate that the LD decay distance can vary in rice depending on the assayed germplasm set. The genetic diversity (π = 1.3 × 10^-3^) of all germplasm in this study was lower than previously described by Huang et al. (2012a) for *Oryza rufipogon* (π = 3.0 × 10^-3^) and *O. sativa* (π = 2.4 × 10^-3^), which may be because all materials used in this study were *japonica* breeding varieties. Genetic diversity was higher in the G1 than in the G2 subpopulation; 91.15% of the germplasm in subpopulation G1 were from Jilin and Liaoning, China, whereas most of the germplasm in subpopulation G2 came from Japan and Heilongjiang, China. Previous studies have shown that *japonica* varieties from Jilin and Liaoning have higher levels of introgression of *indica* consanguinity from the breeding process than *japonica* varieties from Japan and Heilongjiang do (Cui et al., 2022). Thus, the genetic diversity of subpopulation G1 was higher due to the introgression of a higher proportion of *indica* consanguinity. Based on Wright’s (1978) interpretation of F_ST_, there are four ranks of genetic differentiation: mild (F_ST_ = 0–0.05), moderate (0.05–0.15), severe (0.15–0.25), and large (> 0.25). F_ST_ was calculated as 0.1386 between G1 and G2, indicating a moderate degree of genetic differentiation between the two subpopulations. This was smaller than the genetic diversity identified by Luong et al. (2021) (F_ST_ = 0.167) in interspecific genetic differentiation of landraces in Northern Vietnam, which may be because the materials in this study were Northern *japonica* germplasm.

### Co-located loci with previously reported salt tolerance QTLs/genes

The salt stress tolerance traits analyzed by GWAS in this study were GP on different days and GI under salt stress. A total of 52 significant loci were located, of which 17 (33%) were novel loci (**Figure 3**, **Table S3**). Among them, the phenotypic contributions of *q5dGP10* and *q7dGP4-4* was more than 11%, which were major QTLs. Notably, *q5dGP11*, *q6dGP11* and *qGI11* were co-location loci. In addition, 67% of all the loci were co-located with previously reported salt tolerant QTLs/genes (**Figure 3**, **Table S3**), it might confirm the reliability of the positioning results of this study.

One was previously mapped by Yang et al. (2009) between chromosome 4 RM335 and RM551 (0.17–0.68 Mb) as a QTL (*QSst4*) for the level of leaf salt damage at the seedling stage. That study used a high-generation bidirectional backcross import line of the *japonica* variety ‘Lemont’ and the *indica* variety ‘Teqing’ as parents, and *QSst4* overlapped with *q7dGP4-1* and *q7dGP4-2* in this study. The QTL *QSkc4b*, which was associated with aboveground K^+^ concentration, was mapped by Yang et al. (2009) between chromosome 4 RM261 and RM417 (6.57–19.42 Mb); that QTL overlapped with *q7dGP4-5* and *q7dGP4-7* in this study. Zang et al. (2008) constructed a BC_2_F_8_ import line using ‘IR64’ (*indica*) and ‘Binam’ (*japonica*) and mapped a QTL (*QSnc6*) associated with Na^+^ concentration in the seedling stage to a region between chromosome 6 RM527 and RM3 (9.86–19.49 Mb). This region overlapped with *q5dGP6*, *q6dGP6-5*, and *q7dGP6-5* in this study. Wang et al. (2012) mapped a QTL, *qDSW6.1*, related to dry bud weight at the seedling stage between chromosome 6 RM6818 and RM6811 (16.58–29.22 Mb) from RILs (F_1:9_) obtained by crossing the salt-tolerant variety ‘Jiucaiqing’ with the salt-sensitive variety ‘IR26’. This region overlapped with *q5dGP6*, *q6dGP6-5*, and *q7dGP6-7* identified in the present study. The QTL *qDRW6*, which was associated with root dry weight at the seedling stage, was mapped between chromosome 6 RM5531 and RM3183 (7.17–12.44 Mb) in a previous study (Wang et al., 2012); that region overlapped with *q6dGP6-1* and *q7dGP6-1* in the present study. In addition, the known salt stress-related gene *OsHSP1* was co-located with the *q6dGP4-1* and *q7dGP4-1* loci identified here. Moon et al. (2014) found that *Arabidopsis* plants overexpressing *OsHSP1* are more sensitive to salt and osmotic stresses.

### Candidate salt tolerance genes

Activation of SOS1 under salt stress requires activation of SOS2 (an serine/threonine-protein kinase) and the establishment of a proton gradient generated by H^+^-ATPase in the plasma membrane (Haruta and Sussman, 2012). The interaction between the plasma membrane Ca^2+^-ATPase and Cry1Ab/c may affect the salt resistance of the transgenic rice line ‘Huahui-1’ (‘HH1’) by reducing Ca^2+^-ATPase activity under salt stress (Fu et al., 2021). The candidate gene *LOC_Os11g29490* identified in the present study encodes a plasma membrane ATPase, and a single A to G mutation (at chr11:17109866) in the exon caused significant changes in GP between different haplotypes. Hap2 is the superior haplotype conferring stronger salt tolerance and has great potential in breeding applications. In the future, CRISPR/Cas9 could be applied to further verify the function of this gene and apply it in molecular breeding of salt-tolerant rice.

Du et al. (2011) found that there are six genes in the inositol 1,3,4-triphosphate 5/6-kinase (ITPK) family in rice, and that optimal expression of *DMS3*/*OsITPK2* is critical in salt tolerance. Bañuelos et al. (2002) found that there are 17 HAK family members in the rice genome. OsHAK5 enhances rice tolerance to salt stress by balancing K/Na (Horie et al., 2011; Yang et al., 2014), and *oshak1* mutants have increased sensitivity to salt stress (Chen et al., 2015). Therefore, the candidate genes *LOC_Os01g27170* (*OsHAK3*) (which encodes a potassium transporter) and *LOC_Os10g42550* (*OsITPK5*) (which encodes an ITPK) identified in this study may also play important roles in rice salt stress tolerance; mutants for homologous genes are known to have phenotypes related to salt tolerance. Further studies are required to understand the specific molecular mechanisms by which these genes confer salt tolerance in rice.

## Conclusion

In the present study, two evaluation indicators were used to assess salt tolerance in rice at the germination stage: GP on different days and GI under salt stress. These were used as traits for GWAS under salt stress, which yielded 52 significant association sites. The phenotypic contribution rate of 29 loci was > 10%. Five previously identified QTLs for salt tolerance overlapped with loci identified in this study, and one known salt stress-related gene (*OsHSP1*) was also detected. Based on gene annotations and data from the literature, three promising candidate genes for salt tolerance were identified: *LOC_Os01g27170* (*OsHAK3*), *LOC_Os10g42550* (*OsITPK5*), and *LOC_Os11g29490*. The results of this study provide a theoretical basis for cloning, functional analysis, and molecular design breeding of salt tolerance in rice at the germination stage.

## Data availability statement

The data presented in the study are deposited in the National Genomics Data Center (NGDC), part of the China National Center for Bioinformation (CNCB), accession number CRA004238.

## Author contributions

DC, LH, ZZ and LG carried out project design and material preparation. CJ and DC contributed to data analyses. XM, BH, WZ and LG contributed to field trials and phenotype collection. CJ, DC and LH contributed to manuscript writing. All authors contributed to the article and approved the submitted version.

## Funding

This work was supported by the National Key Research and Development Program of China (2021YFD1200500), HAAFS Science and Technology Innovation Special Project (No. 2022KJCXZX-BHS-1), Basic Research Funds of Hebei Academy of Agriculture and Forestry Sciences, the National Natural Sciences Foundation (31670326), CAAS Science and Technology Innovation Program, National Crop Germplasm Resources Center (NCGRC-2021-02).

## Acknowledgments

We thank the National Medium term Genebank, Institute of Crop Sciences, Chinese Academy of Agricultural Sciences for providing the rice seeds.

## Conflict of interest

The authors declare that the research was conducted in the absence of any commercial or financial relationships that could be construed as a potential conflict of interest.

## Publisher’s note

All claims expressed in this article are solely those of the authors and do not necessarily represent those of their affiliated organizations, or those of the publisher, the editors and the reviewers. Any product that may be evaluated in this article, or claim that may be made by its manufacturer, is not guaranteed or endorsed by the publisher.

## Supplementary material

Supplementary Figure 1 ΔK plot for K = 2–9.

Supplementary Figure 2 Average germination potential (GP) on different days under treatment and control. GP on day 3 (3dGP). GP on day 4 (4dGP). GP on day 5 (5dGP). GP on day 6 (6dGP). GP on day 7 (7dGP). ***p* < 0.01 (Student’s *t*-test).

Supplementary Figure 3 Distribution of germination potential (GP) on different days and germination index (GI) under salt stress. (**A**) GP on day 3 (3dGP). (**B**) GP on day 4 (4dGP). (**C**) GP on day 5 (5dGP). (**D**) GP on day 6 (6dGP). (**E**) GP on day 7 (7dGP). (**F**) GI.

Supplementary Figure 4 Average GP on days 3 through 7 under salt stress.

Supplementary Figure 5 Results for Q-Q plot of germination potential (GP) on different days and germination index (GI) under salt stress. (**A**) GP on day 3 (3dGP). (**B**) GP on day 4 (4dGP). (**C**) GP on day 5 (5dGP). (**D**) GP on day 6 (6dGP). (**E**) GP on day 7 (7dGP). (**F**) GI.
