# Supplementary material for: Candidate gene discovery for salt tolerance in rice (Oryza sativa L.) at the germination stage based on genome-wide association study
Source: Front Plant Sci. 2022 Nov 1;13:1010654. doi: 10.3389/fpls.2022.1010654 (PMC9664195; doi:10.3389/fpls.2022.1010654)
Supplement: Supplementary Figure 1 — ΔK plot for K = 2–9. [file DataSheet_2.pdf]

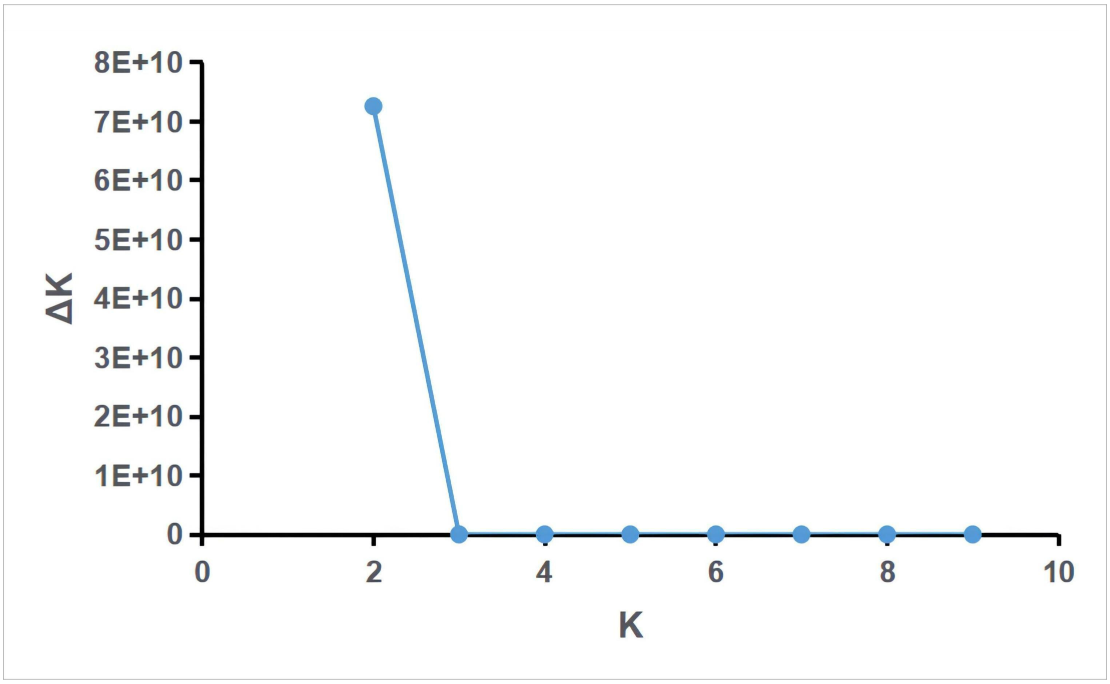

Figure S1 ΔK plot for K = 2–9.

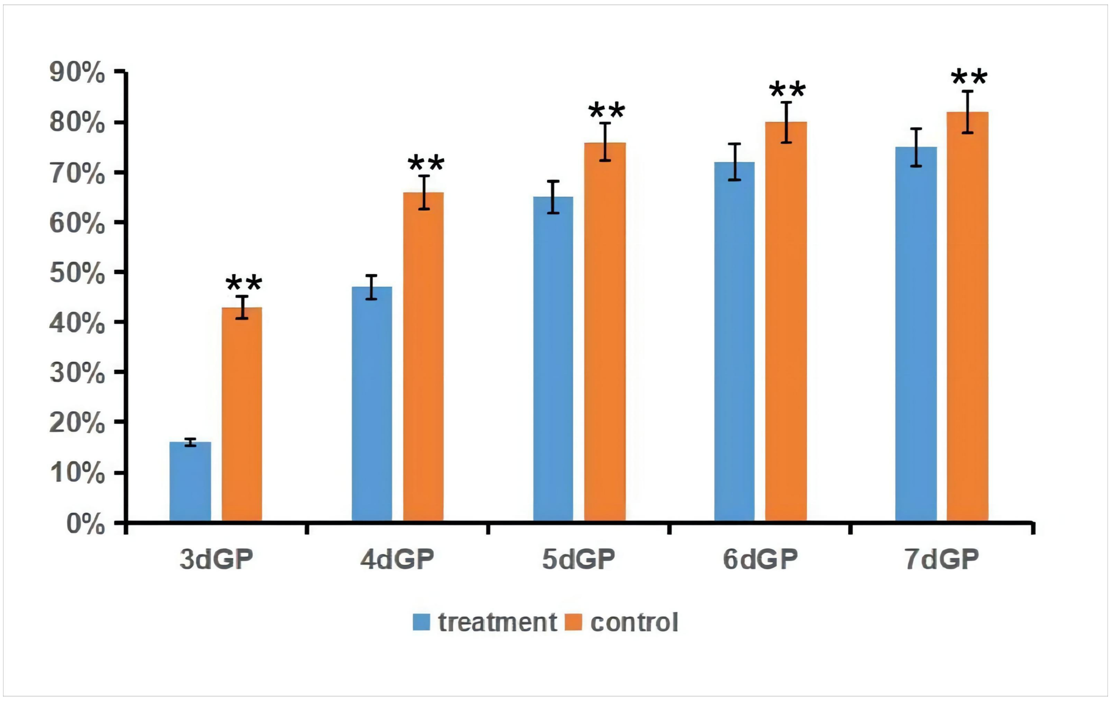

Figure S2 Average germination potential (GP) on different days under treatment and control. GP on day 3 (3dGP). GP on day 4 (4dGP). GP on day 5 (5dGP). GP on day 6 (6dGP). GP on day 7 (7dGP). **$p < 0.01$ (Student's *t*-test).

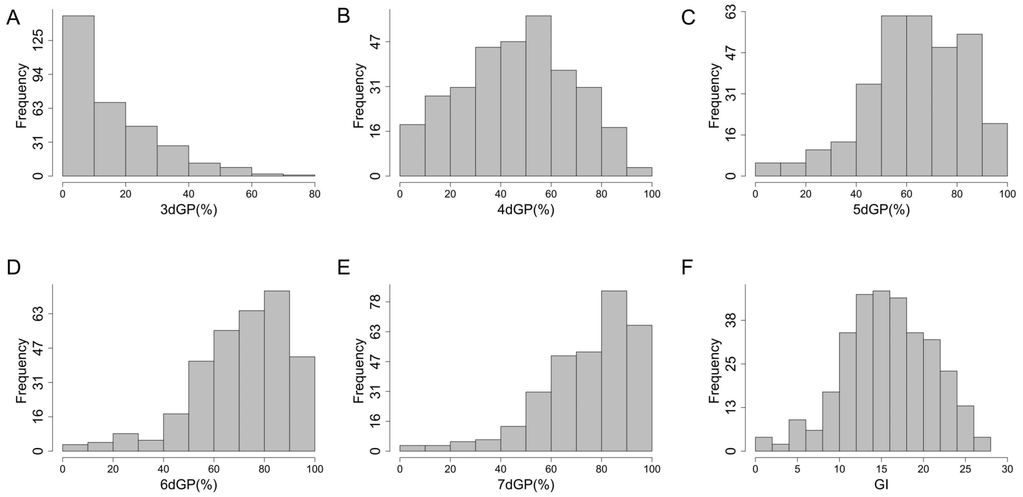

Figure S3 Distribution of germination potential (GP) on different days and germination index (GI) under salt stress. (**A**) GP on day 3 (3dGP). (**B**) GP on day 4 (4dGP). (**C**) GP on day 5 (5dGP). (**D**) GP on day 6 (6dGP). (**E**) GP on day 7 (7dGP). (**F**) GI.

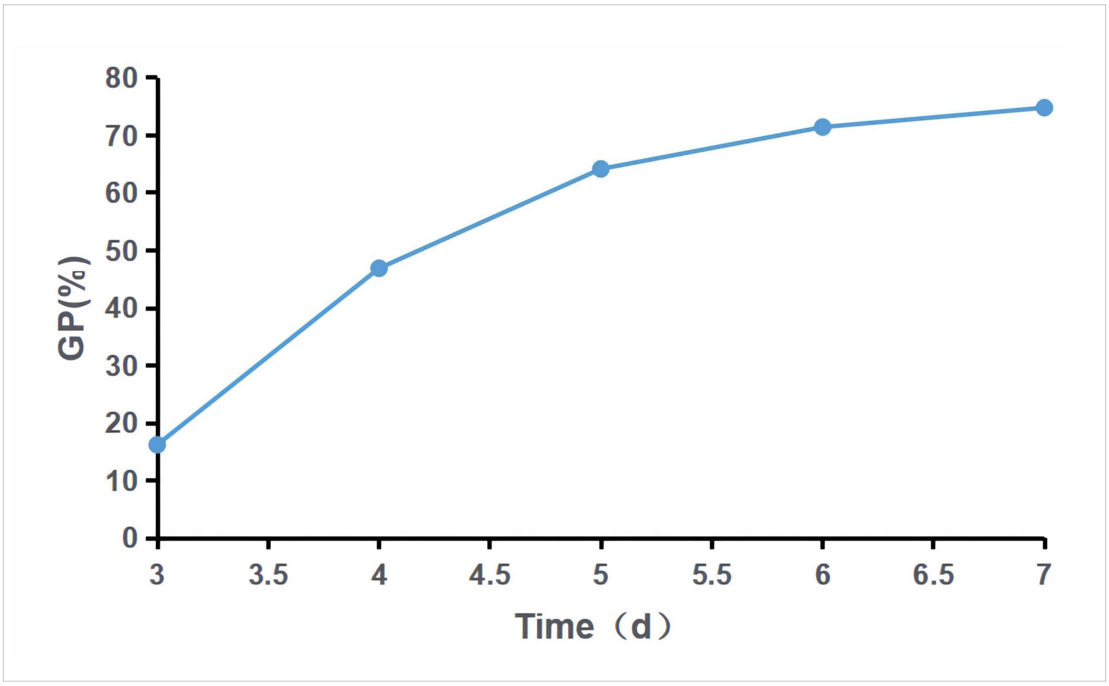

Figure S4 Average GP on days 3 through 7 under salt stress.

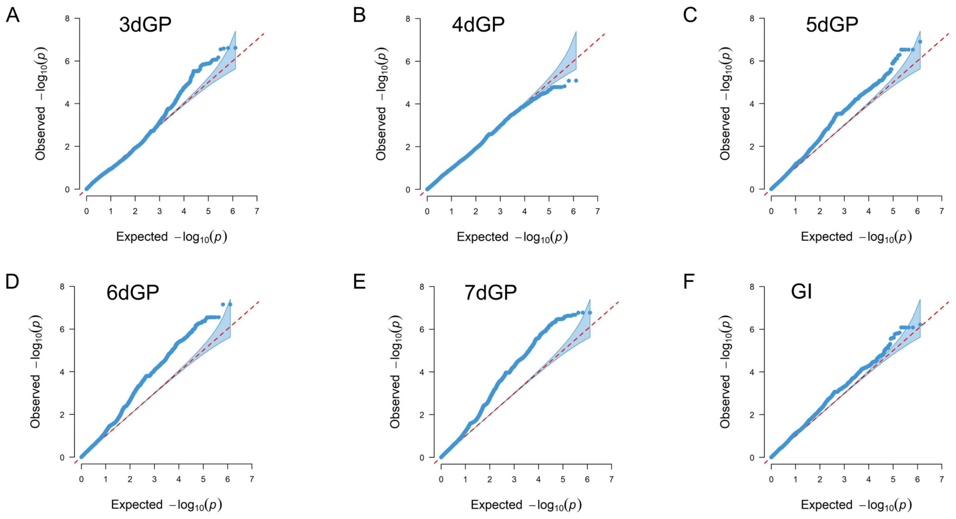

Figure S5 Results for Q-Q plot of germination potential (GP) on different days and germination index (GI) under salt stress. (**A**) GP on day 3 (3dGP). (**B**) GP on day 4 (4dGP). (**C**) GP on day 5 (5dGP). (**D**) GP on day 6 (6dGP). (**E**) GP on day 7 (7dGP). (**F**) GI.
